# Supplementary figures and images for: A Statistical Framework for Joint eQTL Analysis in Multiple Tissues
Source: PLoS Genet. 2013 May 9;9(5):e1003486. doi: 10.1371/journal.pgen.1003486 (PMC3649995; doi:10.1371/journal.pgen.1003486)

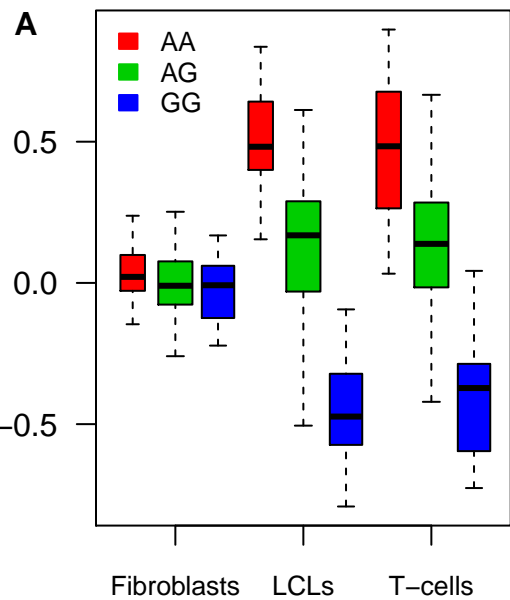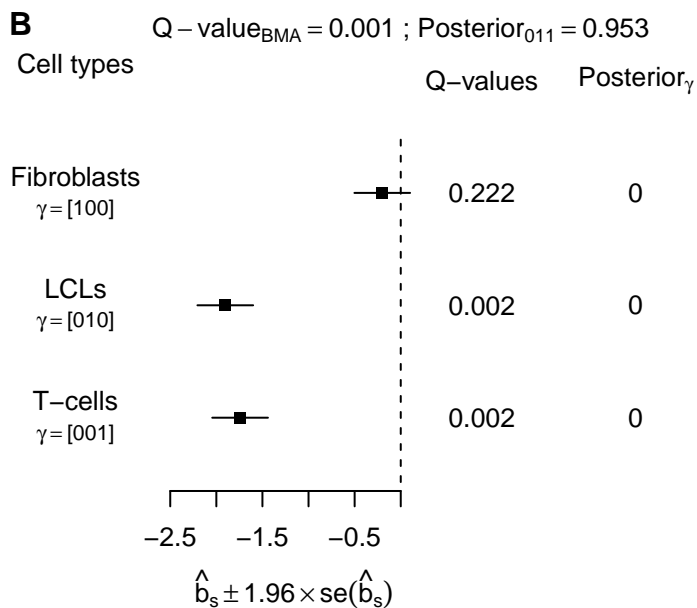

Supplement: Figure S1 — Example of a strong, tissue-specific eQTL. A. Boxplots of the PC-corrected expression levels from gene ANKDD1A (Ensembl id ENSG00000166839) in all three cell types, color-coded by genotype class at SNP rs1628955. B. Forest plot of estimated standardized effect sizes of this eQTL. The posterior probability for configuration (0,1,1) is above 0.95, indicating that this eQTL is very likely to be active in LCLs and T-cells but not in fibroblasts. (PDF) [file pgen.1003486.s001.pdf]

**Histogram of effect sizes  
when simulated according to the grid A**

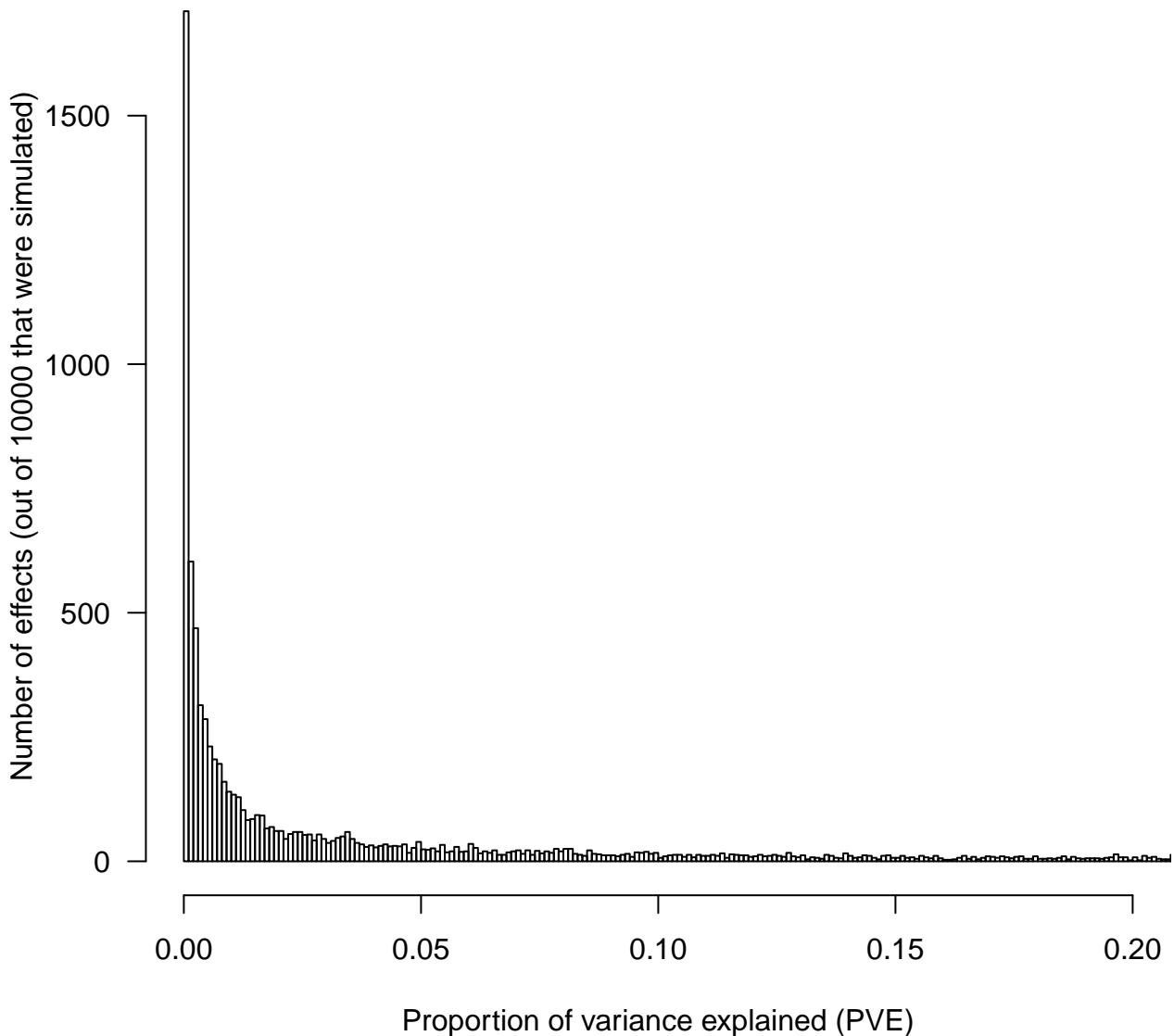

Supplement: Figure S2 — Histogram of effect sizes when simulated according to the grid A. (PDF) [file pgen.1003486.s002.pdf]

Number of genes (out of 5012)

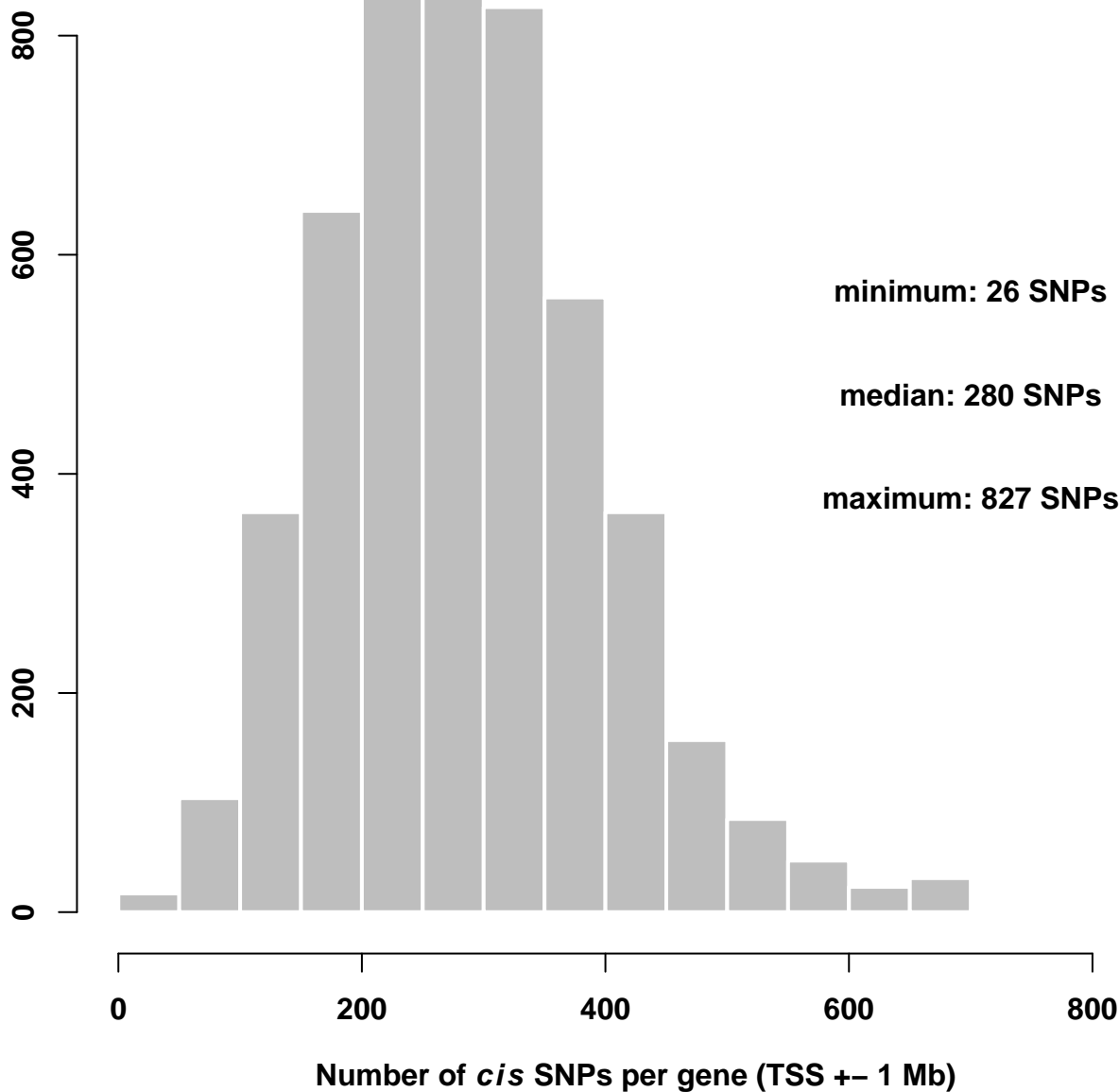

Supplement: Figure S3 — Histogram of the number of SNPs in the cis region of each gene for the data set from Dimas et al. (PDF) [file pgen.1003486.s003.pdf]
