# Supplementary material for: A Statistical Framework for Joint eQTL Analysis in Multiple Tissues
Source: PLoS Genet. 2013 May 9;9(5):e1003486. doi: 10.1371/journal.pgen.1003486 (PMC3649995; doi:10.1371/journal.pgen.1003486)
Supplement: Text S1 — Supplementary methods and results. (PDF) [file pgen.1003486.s004.pdf]

Supplementary text S1 for "A statistical framework for joint eQTL analysis in multiple tissues"

Timothée Flutre, Xiaoquan Wen, Jonathan Pritchard, Matthew Stephens

Contents

1 Computational algorithm for fitting hierarchical model **1**
1.1 Notations . . . 1
1.2 Maximum Likelihood Inference based on EM algorithm . . . 2

2 Supplements for the simulations **4**
2.1 Simulate eQTL data via the proportion of variance explained . . . 4
2.2 Implement the ANOVA/LR model in R . . . 4
2.3 Calculate the empirical FDR from simulated eQTL data . . . 5

3 Supplements for the analysis of the Dimas *et al.* data set **5**
3.1 Choice of the grid for the average effect size . . . 5
3.2 Hierarchical model fed with Bayes Factors from residuals . . . 6
3.3 Configuration proportions from all genes without removing expression PCs . . . 7
3.4 Running times . . . 7

1 Computational algorithm for fitting hierarchical model

For the hierarchical model described in the main text, our primary interest is making inference on the parameter set $\Theta = (\pi_0, \boldsymbol{\eta}, \boldsymbol{\lambda})$. Here, we give details of an algorithm for inferring Θ, via maximum likelihood estimation based on the EM algorithm.

1.1 Notations

Throughout this section, we adopt the following additional notations. For gene k, we use a latent binary indicator z_k to denote if there is any eQTL in its *cis*-region for any tissue type, in particular,

$$\Pr(z_k = 1) = 1 - \pi_0; \tag{1}$$

We use a latent random indicator m_k-vector $\boldsymbol{s}_k$ to denote the true eQTL SNP conditional on $z_k = 1$ and let s_{kp} denote the p-th entry of $\boldsymbol{s}_k$. The "one *cis* eQTL per gene" assumption restricts $\boldsymbol{s}_k$ can have at most one entry equaling 1 (with the remaining entries being 0). By this definition,

$$\Pr(\boldsymbol{s}_k = \mathbf{0} | z_k = 0) = 1, \tag{2}$$

and we also make the simplifying assumption that

$$\Pr(s_{kp} = 1 | z_k = 1) = \frac{1}{m_k}. \tag{3}$$

Furthermore, for gene k and SNP p, we index all configurations and use a $(2^S - 1)$-dimension latent indicator vector $\boldsymbol{c}_{kp}$ to denote the actual configuration for the gene–SNP pair. In case the SNP is not an eQTL,

$$\Pr(\boldsymbol{c}_{kp} = \mathbf{0} | s_{kp} = 0) = 1. \tag{4}$$

Otherwise, we assume the jth configuration is active with prior probability

$$\Pr(c_{kpj} = 1 | s_{kp} = 1) = \eta_j. \tag{5}$$

Joining the column vectors $\boldsymbol{c}_{kp}$ for all m_k SNPs, we obtain a latent $(2^S - 1) \times m_k$ random matrix C_k. Finally, we use the latent L-vector $\boldsymbol{w}_{kp}$ indicate the actual prior effect size for active tissue types for the pair of gene k and SNP p. The m-th entry of the indicator is denoted by w_{kpm}, for which we assume prior probability

$$\Pr(\boldsymbol{w}_{kp} = \boldsymbol{0} | s_{kp} = 0) = 1, \tag{6}$$

and

$$\Pr(w_{kpm} = 1 | s_{kp} = 1) = \lambda_m. \tag{7}$$

Joining the column vectors $\boldsymbol{w}_{kp}$ for all m_k SNPs, we obtain a latent $L \times m_k$ random matrix W_k

1.2 Maximum Likelihood Inference based on EM algorithm

In the maximum likelihood framework, we treat latent variables $z_k, \boldsymbol{s}_k, \boldsymbol{c}_k$ and $\boldsymbol{w}_k, k = 1, \ldots, G$ as missing data and apply the EM algorithm.

For a total number of G genes, let $\boldsymbol{z} = (z_1, \ldots, z_G), \boldsymbol{S} = (\boldsymbol{s}_1, \ldots, \boldsymbol{s}_G), \boldsymbol{C} = (C_1, \ldots, C_G)$ and $\boldsymbol{W} = (W_1, \ldots W_G)$ denote the complete collection of latent variables. Let $\boldsymbol{Y} = (\boldsymbol{Y}_1, \ldots \boldsymbol{Y}_G)$ and $\boldsymbol{G} = (G_1, \ldots, G_G)$ denote the complete set of observed data. Based on the hierarchical model described in previous section, we can write out the complete data log-likelihood as follows,

$$\begin{aligned}
\log\ & p(\boldsymbol{Y}, \boldsymbol{z}, \boldsymbol{S}, \boldsymbol{C}, \boldsymbol{W} | \boldsymbol{G}, \Theta) = \\
& \sum_k (1 - z_k) \log \pi_0 + \sum_k z_k \log(1 - \pi_0) \\
& + \sum_{k,p} z_k s_{kp} \log \frac{1}{m_k} + \sum_{k,p,j} z_k s_{kp} c_{kpj} \log \eta_j + \sum_{k,p,m} z_k s_{kp} w_{kpm} \log \lambda_m \\
& + \sum_{k,p,j,m} z_k s_{kp} c_{kpj} w_{kpm} \cdot \mathrm{BF}_{kpjm} + \sum_k \log p_k^0.
\end{aligned} \tag{8}$$

In (8), p_k^0 denotes the likelihood of the null model for gene k, i.e.,

$$p_k^0 := p(\boldsymbol{Y}_k | z_k = 0) \tag{9}$$

and

$$\mathrm{BF}_{kpjm} = \frac{P(\boldsymbol{Y}_k | z_k = 1, s_{kp} = 1, c_{kpj} = 1, w_{kpm} = 1, \boldsymbol{G}_k, \Theta)}{p_k^0} \tag{10}$$

is the Bayes Factor (pre-)computed for a fully specified alternative model.

The EM algorithm searches for maximum likelihood estimate of Θ, by iteratively performing an expectation (E) step and a maximization (M) step.

In the E-step, for the t-th iteration, we evaluate the expectation of complete data log-likelihood (8) conditional on current estimate of parameter $\Theta^{(t)}$, $\boldsymbol{G}$ and $\boldsymbol{Y}$. The computation is straightforward, for example,

$$\begin{aligned}
\mathrm{E}(z_k | \boldsymbol{Y}_k, \boldsymbol{G}_k, \Theta^{(t)}) &= \Pr(z_k = 1 | \boldsymbol{Y}_k, \boldsymbol{G}_k, \Theta^{(t)}) \\
&= \frac{\Pr(z_k = 1 | \Theta^{(t)}) \cdot p(\boldsymbol{Y}_k | z_k = 1, \boldsymbol{G}_k, \Theta^{(t)})}{p(\boldsymbol{Y}_k | \boldsymbol{G}_k, \Theta^{(t)})} \\
&= \frac{(1 - \pi_0^{(t)}) \mathrm{BF}_k^{(t)}}{\pi_0^{(t)} + (1 - \pi_0^{(t)}) \mathrm{BF}_k^{(t)}},
\end{aligned} \tag{11}$$

similarly,

$$\mathrm{E}(z_k s_{kp}|\boldsymbol{Y}_k, \boldsymbol{G}_k, \Theta^{(t)}) = \frac{(1-\pi_0^{(t)})\frac{1}{m_k}\mathrm{BF}_{kp}^{(t)}}{\pi_0^{(t)} + (1-\pi_0^{(t)})\mathrm{BF}_k^{(t)}}, \tag{12}$$

$$\mathrm{E}(z_k s_{kp} c_{kpj} w_{kpm}|\boldsymbol{Y}, \boldsymbol{G}, \Theta^{(t)}) = \frac{(1-\pi_0^{(t)})\frac{1}{m_k}\eta_j^{(t)}\lambda_m^{(t)}\mathrm{BF}_{kpjm}}{\pi_0^{(t)} + (1-\pi_0^{(t)})\mathrm{BF}_k^{(t)}}, \tag{13}$$

where

$$\begin{aligned}\mathrm{BF}_k^{(t)} &= \frac{p(\boldsymbol{Y}_k|z_k = 1, \boldsymbol{G}_k, \Theta^{(t)})}{p_k^0} \\ &= \sum_{p,j,m} \frac{1}{m_k}\eta_j^{(t)}\lambda_m^{(t)}\mathrm{BF}_{kpjm},\end{aligned} \tag{14}$$

and

$$\begin{aligned}\mathrm{BF}_{kp}^{(t)} &= \frac{p(\boldsymbol{Y}_k|z_k = 1, s_{kp} = 1, \boldsymbol{G}_k, \Theta)}{p_k^0} \\ &= \sum_{j,m} \eta_j^{(t)}\lambda_m^{(t)}\mathrm{BF}_{kpjm},\end{aligned} \tag{15}$$

In the M-step, we find a new set of estimates, $\Theta^{(n+1)}$, by maximizing the conditional expectation $\mathrm{E}\left(\log p(\boldsymbol{Y}, \boldsymbol{z}, \boldsymbol{S}, \boldsymbol{C}, \boldsymbol{W}|\boldsymbol{G}, \Theta)|\boldsymbol{Y}, \boldsymbol{G}, \Theta^{(t)}\right)$. In this case, the simultaneous maximization can be performed analytically. In particular,

$$\pi_0^{(t+1)} = \frac{1}{g}\sum_{k=1}^{g} \frac{\pi_0^{(t)}}{\pi_0^{(t)} + (1-\pi_0^{(t)})\mathrm{BF}_k^{(t)}}, \tag{16}$$

$$\eta_j^{(t+1)} = \frac{\sum_{k,p,m} \frac{\gamma_{kp}^{(t)}\lambda_m^{(t)}\mathrm{BF}_{kpjm}}{\pi_0^{(t)}+(1-\pi_0^{(t)})\mathrm{BF}_k^{(t)}} \cdot \eta_j^{(t)}}{\sum_{j'}\left(\sum_{k,p,m} \frac{\gamma_{kp}^{(t)}\lambda_m^{(t)}\mathrm{BF}_{kpj'm}}{\pi_0^{(t)}+(1-\pi_0^{(t)})\mathrm{BF}_k^{(t)}} \cdot \eta_{j'}^{(t)}\right)}, \tag{17}$$

and

$$\lambda_m^{(t+1)} = \frac{\sum_{k,p,j} \frac{\gamma_{kp}^{(t)}\eta_j^{(t)}\mathrm{BF}_{kpjm}}{\pi_0^{(t)}+(1-\pi_0^{(t)})\mathrm{BF}_k^{(t)}} \cdot \lambda_m^{(t)}}{\sum_{m'}\left(\sum_{k,p,j} \frac{\gamma_{kp}^{(t)}\eta_j^{(t)}\mathrm{BF}_{kpjm'}}{\pi_0^{(t)}+(1-\pi_0^{(t)})\mathrm{BF}_k^{(t)}} \cdot \lambda_{m'}^{(t)}\right)}. \tag{18}$$

Typically, we initiate the EM algorithm by setting $\Theta^{(0)}$ to some random values and running iterations until some pre-defined convergence threshold is met (In practice, we monitor the increase of the the log-likelihood function between successive iterations, and stop the iterations as the increment becomes sufficiently small.).

We construct profile likelihood confidence intervals for estimated parameters. For example, a $(1-\alpha)\%$ profile likelihood confidence set for π_0 is built as

$$\{\pi_0 : \log p(\boldsymbol{Y}|\pi_0, \hat{\boldsymbol{\eta}}, \hat{\boldsymbol{\lambda}}, \boldsymbol{G}) > \log p(\boldsymbol{Y}|\hat{\pi}_0, \hat{\boldsymbol{\eta}}, \hat{\boldsymbol{\lambda}}, \boldsymbol{G}) - \frac{1}{2}Z_{(1-\alpha)}^2\}, \tag{19}$$

where $\hat{\pi}_0, \hat{\boldsymbol{\eta}}, \hat{\boldsymbol{\lambda}}$ are MLEs obtained from the EM algorithm.

2 Supplements for the simulations

2.1 Simulate eQTL data via the proportion of variance explained

For a given gene-SNP pair at a time, we simulate data in S tissues according to a particular configuration. In a given tissue $s \in \{1, \dots, S\}$ for which the SNP is an eQTL ($\beta_s \neq 0$), let's define the proportion of variance in phenotype explained by the genotype:

$$PVE_s(\beta_s, \sigma_s) = \frac{V(X\beta_s)}{V(X\beta_s) + \sigma_s^2}$$

When working with standardized effect sizes $b_s = \beta_s / \sigma_s$:

$$PVE_s = \frac{V(Xb_s)}{V(Xb_s) + 1}$$

As stated elsewhere ([1]), we approximate the expectation of the PVE via a ratio of expectations, noted h:

$$h = \frac{E[V(Xb_s)]}{E[V(Xb_s)] + 1}$$

We assume that the genotypes are drawn from a Binomial distribution with parameters 2 and f, the minor allele frequency, so that $E[V(X)] = 2f(1-f)$. Moreover, as we assume $b_s|\bar{b} \sim N(\bar{b}, \phi^2)$ and $\bar{b} \sim N(0, \omega^2)$, the marginal effect size is $b_s \sim N(0, \phi^2 + \omega^2)$. We can hence approximate b_s^2 by its variance. Therefore:

$$h = \frac{(\phi^2 + \omega^2) \times 2f(1-f)}{(\phi^2 + \omega^2) \times 2f(1-f) + 1}$$

By fixing h (e.g. 20%) as well as the minor allele frequency (e.g. 30%), we obtain:

$$\phi^2 + \omega^2 = \frac{h}{(1-h) \times 2f(1-f)}$$

Now if we fix the heterogeneity in effect sizes (e.g. $\phi^2/(\phi^2 + \omega^2) = 20\%$), we can deduce ϕ^2 and then ω^2. We can hence draw $\bar{b}$ and then each $b_s|\bar{b}$.

Once we have them, it is straightforward to simulate the phenotype of the ith individual in the sth tissue:

$$y_{is} = b_s \sigma_s g_i + \mathcal{N}(0, \sigma_s^2)$$

with σ_s being fixed at 1 for instance.

2.2 Implement the ANOVA/LR model in R

For each gene-SNP pair, the expression levels from all N individuals in all S tissues are recorded into a vector `y` of length $N \times S$. The genotypes are appropriately repeated S times into a vector `xg`, and the tissue indicators are appropriately recorded into a vector `xs`. We can then use the ANOVA/LR model to test if there is an effect of the genotype with the following commands:

```
m1 <- lm(y ~ xs)
m2 <- lm(y ~ xs * xg)
pval <- anova(m1, m2)[[6]][2]
```

2.3 Calculate the empirical FDR from simulated eQTL data

For a simulated data set of G gene-SNP pairs, let z_g be the test statistic of a given pair with $g = 1, \ldots, G$. For the tissue-by-tissue method, we take as test statistic the minimum p-value across tissues. For the Bayesian method, the test statistic is the Bayes Factor. For the ANOVA/LR, the test statistic is the p-value from the F test comparing the null model (tissue indicator only) with the unconstrained alternative (tissue indicator, genotype and their interaction).

All gene-SNP pairs can be classified as in the following table ([2]):

	Called eQTL	Not called	Total
True null	F	G_0 - F	G_0
True eQTL	T	G_1 - T	G_1
Total	S	G - S	G

As we simulate data, we know which pairs are true eQTLs. By fixing the empirical false discovery rate (FDR_e) at 5%, we can find the corresponding cutoff c on the test statistics, and from there calculate the true positive rate (TPR) at this cutoff:

$TPR(c) = T(c)/G_1$ with c such that $FDR_e(c) = F(c)/S(c) = 0.05$.

The following algorithm describes how to iteratively find the cutoff c corresponding to the 5% empirical FDR:

Data: test statistics $z_1, \ldots, z_G$

if *p-values* **then**
  sort in increasing order: $z_{(1)} \leq \ldots \leq z_{(G)}$
else if *Bayes Factors* **then**
  sort in decreasing order: $z_{(1)} \geq \ldots \geq z_{(G)}$

foreach *gene-SNP pair* $g \leftarrow 1$ **to** G **do**
  $c \leftarrow z_{(g)}$
  $s \leftarrow$number of called eQTLs at this cutoff c
  $f \leftarrow$number of false positives among them
  $fdr \leftarrow f/s$
  if $fdr \geq 5\%$ **then**
    $t \leftarrow$number of true positives among the called eQTLs
    $tpr \leftarrow t/s$
    exit

Between different methods, the empirical FDRs will always be 5% (or slightly higher) but the TPRs and FPRs will be different, which allows us to compare the performance of the methods.

3 Supplements for the analysis of the Dimas *et al.* data set

3.1 Choice of the grid for the average effect size

The grid noted A in the Methods section of the main text corresponds to an expected (average) effect size, but even one grid point would allow for a range of actual effect sizes (normally distributed, with the specified variance); thus even large values of A allow for some effects that are very close to 0. Multiple grid points are helpful to allow for a longer-tailed distribution of effects than a single normal. The most

important thing is that the grid of A values is broad enough to covers the range of effect sizes detectable in the data — results are relatively insensitive to the inclusion of a few grid points that are bigger or smaller than can be detected, particularly in the hierarchical model where grid weights are estimated from the data — so maybe best to err on the side of a grid spanning too large a range. The grid we used reflects this, and was chosen to span (more than) the full range of effect sizes we think are plausibly detectable; as a result it would probably be unnecessary to add more smaller values even in larger studies.

To illustrate, we simulated PVE values using our grid (with equal weight on each grid point), for a SNP with frequency 20% (see R code below). The median simulated PVE was 0.017. The 5th and 95th percentiles were $[8.7 \times 10^{-5}, 0.54]$, which we view as spanning a range of impossible-to-detect to unrealistically large effects (see also supplementary figure S2). This range means that, for a SNP at frequency 0.2, equal weight on this grid implies that 90% of eQTLS will explain between 8.7×10^{-5} and 0.54 of the total variance in expression.

```
set.seed(100)
nb.eqtls <- 10000
sdbeta <- sample(c(0.1,0.2,0.4,0.8,1.6), nb.eqtls, replace=TRUE)
beta <- rnorm(nb.eqtls, sd=sdbeta)
f <- 0.2
vg <- beta^2 * 2*f*(1-f)
pve <- vg / (1+vg)
quantile(pve, probs=c(0.01,0.05,0.1,0.5,0.95))
hist(pve, xlim=c(0,0.2), breaks=1000)
```

3.2 Hierarchical model fed with Bayes Factors from residuals

First we computed the Bayes Factors for each combination of grid values and configurations, one gene-SNP pair at a time. Second, for each gene, we regressed out the effect of its best SNP, and we recomputed the Bayes Factors for the remaining SNPs using the residuals as phenotypes. Third, we launched the hierarchical model with only the Bayes Factors obtained from the residuals.

If the "at most one eQTL per gene" assumption is reasonable for this data set, we would expect the estimated π_0 to be very high (meaning that the vast majority of genes have no eQTL), the lowest grid value to have the highest probability (meaning that the effect sizes are very small), and the credible intervals for the configurations to be very large (corresponding to high uncertainty).

This is indeed what we observe:

π_0: 0.963 [0.946, 1.000]

Grid value (ϕ^2, ω^2)	Posterior mean	95% credible interval
(0.01, 0.01)	0.930	[0.543, 1.000]
(0.01, 0.04)	0.070	[0.000, 0.459]
(0.01, 0.16)	0.000	[0.000, 0.107]
(0.01, 0.64)	0.000	[0.000, 0.036]
(0.01, 2.56)	0.000	[0.000, 0.019]

Configuration	Posterior mean	95% credible interval
100	0.316	[0.000, 1.000]
010	0.330	[0.000, 1.000]
001	0.027	[0.000, 0.535]
110	0.250	[0.000, 1.000]
101	0.020	[0.000, 0.442]
011	0.029	[0.000, 0.535]
111	0.028	[0.000, 0.400]

3.3 Configuration proportions from all genes without removing expression PCs

Similarly to what was done in the first analysis of this data set ([3]), we also analyzed the data set comprising all 12,046 genes, i.e. without pre-selecting genes robustly expressed in all three tissues, and without removing expression PCs. Here are the configuration proportions estimated by the EM algorithm:

Configuration	Hierarchical model
F-L-T	0.793 [0.722, 0.878]
L-T	0.071 [0.030, 0.129]
F-L	0.000 [0.000, 0.015]
F-T	0.000 [0.000, 0.015]
F	0.052 [0.000, 0.109]
L	0.058 [0.016, 0.115]
T	0.025 [0.000, 0.068]

They are thus qualitatively similar to those obtained on the subset of genes robustly expressed in all three tissues and after having removed PCs (table 1 of the main text).

3.4 Running times

After our preprocessing, the data set from Dimas *et al.* totalizes 5012 genes and 418,477 SNPs. Focusing on the *cis* region $\pm$ 1 Mb around the transcription start site of each gene yields 1,436,869 gene-SNP pairs to test for association.

The first step of our approach is to compute the Bayes Factors for all configurations and grid values. As each gene-SNP pair can be analyzed in parallel, we splitted the genes in 101 batches (i.e. $\approx$ 50 genes per batch). Our first program, `eqtlbma`, spent a total of 36 minutes to calculate all Bayes Factors. The second step then uses all Bayes Factors when fitting the hierarchical model via an EM algorithm. On this data set, our second program, `hm`, ran in 2 hours.

The `eqtlbma` program can also perform permutations per gene. Each job ran in less than 8 hours when BF_{BMA} is the test statistic, and in less than 6 hours when $\text{BF}_{\text{BMAlite}}$ is the test statistic. In this case (3 tissues), BF_{BMA} is averaged over 7 configurations whereas $\text{BF}_{\text{BMAlite}}$ is averaged over 4 configurations. This small difference between the number of configurations over which each test statistic is averaged over explains why the running time is only 25% less with $\text{BF}_{\text{BMAlite}}$ compare to BF_{BMA}, but the gain will increase with the number of tissues.

Both programs are written in C++. The `eqtlbma` measures running times using the `CLOCKS_PER_SEC` macro from the C library. For the `hm` program, we measured running time as the duration between the time at which the execution started and at which it ended.

References

1. Guan Y, Stephens M (2011) Bayesian variable selection regression for genome-wide association studies, and other Large-Scale problems. Annals of Applied Statistics .

2. Storey JD, Tibshirani R (2003) Statistical significance for genomewide studies. Proceedings of the National Academy of Sciences of the United States of America 100: 9440–9445.

3. Dimas AS, Deutsch S, Stranger BE, Montgomery SB, Borel C, et al. (2009) Common regulatory variation impacts gene expression in a cell type-dependent manner. Science 325: 1246–1250.
